# From fat to fire: The lipid–inflammasome connection

**DOI:** 10.1111/imr.13403

**Published:** 2024-09-27

**Authors:** Paras K. Anand

**Affiliations:** ^1^ Department of Infectious Disease, Faculty of Medicine Imperial College London London UK

**Keywords:** cholesterol, cholesterol trafficking, FASN, fatty acid biosynthesis, inflammasomes, lipid metabolism, lipids, NLRP3, phosphatidylinositol

## Abstract

Inflammasomes are multiprotein complexes that play a crucial role in regulating immune responses by governing the activation of Caspase‐1, the secretion of pro‐inflammatory cytokines, and the induction of inflammatory cell death, pyroptosis. The inflammasomes are pivotal in effective host defense against a range of pathogens. Yet, overt activation of inflammasome signaling can be detrimental. The most well‐studied NLRP3 inflammasome has the ability to detect a variety of stimuli including pathogen‐associated molecular patterns, environmental irritants, and endogenous stimuli released from dying cells. Additionally, NLRP3 acts as a key sensor of cellular homeostasis and can be activated by disturbances in diverse metabolic pathways. Consequently, NLRP3 is considered a key player linking metabolic dysregulation to numerous inflammatory disorders such as gout, diabetes, and atherosclerosis. Recently, compelling studies have highlighted a connection between lipids and the regulation of NLRP3 inflammasome. Lipids are integral to cellular processes that serve not only in maintaining the structural integrity and subcellular compartmentalization, but also in contributing to physiological equilibrium. Certain lipid species are known to define NLRP3 subcellular localization, therefore directly influencing the site of inflammasome assembly and activation. For instance, phosphatidylinositol 4‐phosphate plays a crucial role in NLRP3 localization to the *trans* Golgi network. Moreover, new evidence has demonstrated the roles of lipid biosynthesis and trafficking in activation of the NLRP3 inflammasome. This review summarizes and discusses these emerging and varied roles of lipid metabolism in inflammasome activation. A deeper understanding of lipid‐inflammasome interactions may open new avenues for therapeutic interventions to prevent or treat chronic inflammatory and autoimmune conditions.

## 1 INTRODUCTION

A key function of the immune system is to sense changes in the immediate environment and respond appropriately.^1^, ^2^, ^3^ The response triggered is often multifaceted, involving the deployment of molecular machinery that challenges infection or injury. This is followed by processes that clear damage and debris aiding efforts to restore physiological equilibrium.^4^, ^5^ One of the main arsenals against any form of perturbation is germline‐encoded pattern‐recognition receptors (PRRs) which sense pathogen‐associated (PAMPs) and danger‐associated molecular patterns (DAMPs).^1^, ^6^, ^7^, ^8^ These PRRs integrate cues and activate downstream pathways to reconstitute homeostasis.

PRRs belong to several distinct families and some of the major ones include the TLRs, C‐type lectin receptors, RIG‐I‐like receptors, and the Nod‐like receptors (NLR).^9^, ^10^ The PRRs are localized either in the cytosol, endosomes, or at the cell surface to recognize threats at different levels of invasion.^11^ NLRs are cytosolic receptors which sense cytosolic breach and have the potential to trigger various effector pathways, including the NF‐kB and MAP‐kinase signaling, interferon production, cell‐autonomous immunity, and inflammasome activation.^2^, ^12^, ^13^, ^14^, ^15^ The activation of the inflammasome, in particular, is an elegant but a tightly regulated process in which a multiprotein complex is assembled.^16^, ^17^ Several NLRs have the ability to assemble an inflammasome.^18^, ^19^ However, the complex assembled by the sensor protein NLRP3, together with the adaptor protein ASC and effector protein Caspase‐1, is the most well studied.^19^, ^20^, ^21^ The NLRP3 sensor protein is composed of three key domains: an N‐terminal pyrin (PYD) domain, a central nucleotide‐binding and oligomerization (NACHT) domain, and a C‐terminal leucine‐rich repeat (LRR) domain.^19^, ^21^ The formation of the NLRP3 inflammasome consists of homotypic interactions with the corresponding domains of ASC and Caspase‐1.^18^, ^22^ During the resting stage, the NLRP3 LRR domain is thought to fold back onto the NACHT domain inhibiting its autoactivation.^23^ Upon activation, the NLRP3 oligomerizes and further recruits ASC, interacting with it through their homotypic PYD domains.^22^, ^24^, ^25^ Subsequently, Caspase‐1 is recruited into the complex through CARD‐CARD domain interactions with ASC resulting in Caspase‐1 autoactivation.

The NLRP3 inflammasome is assembled in response to a range of stimuli, making it pivotal in a variety of inflammatory and autoimmune diseases.^13^, ^26^ In contrast to other inflammasomes, the activation of NLRP3 requires two distinct steps, known as the priming and the activation steps.^16^, ^27^ Intriguingly, the priming step involves coordination with membrane‐localized TLRs for upregulation of NLRP3 expression and synthesis of pro‐IL‐1β.^28^, ^29^ Furthermore, TLR priming also plays a role in post‐translationally modifying the inflammasome components.^30^ A critical role for ubiquitination, phosphorylation, and palmitoylation in inflammasome activation has been identified in recent years.^31^, ^32^ The nature of the NLRP3 activating stimuli ranges from microbial pore‐forming toxins to endogenous (such as ATP, cholesterol, and uric acid crystals) and environmental stimuli (such as asbestos, silica, and alum).^27^, ^33^ Given the wide variety in the nature of these stimuli, a common cellular event has been hypothesized to mediate inflammasome activation. Separate studies have identified lysosomal damage, generation of reactive oxygen species, potassium efflux, and mitochondrial dysfunction as the putative upstream events triggering inflammasome assembly.^34^, ^35^, ^36^ However, K+ efflux has been demonstrated to be the unifying mechanism common to all NLRP3 stimuli. Nonetheless, activation stimuli that trigger NLRP3 formation independently of K^+^ efflux are known.^37^ Additionally, it remains unclear whether K^+^ efflux is directly sensed by NLRP3 sensor protein.

Inflammasome assembly triggers Caspase‐1 activation, which processes several downstream substrates, including Gasdermin D (GSDMD). Upon activation, GSDMD translocates to the plasma membrane, forming pores that lead to membrane depolarization and ultimately cell rupture. These pores also serve as conduits for secretion of Caspase‐1 processed cytokines, IL‐1β and IL‐18, mechanistically connecting inflammasome activation to cytokine secretion. The activation of the NLRP3 inflammasome may be additionally facilitated by accessory molecules, which vary depending on the specific context. FADD and caspase‐8 promote both the priming and activation of the NLRP3 inflammasome, though caspase‐8 may be inhibitory under certain conditions.^38^, ^39^ Moreover, Nima‐related kinase 7 (NEK7), a protein kinase required for mitotic spindle formation, has been reported to be a component of the NLRP3 inflammasome complex.^40^, ^41^ However, iPSC‐derived human macrophages form an NLRP3 inflammasome independently of NEK7.^42^ This highlights the complexities and the diverse, context‐dependent mechanisms involved in the regulation of the NLRP3 inflammasome.

Notably, overt activation of the NLRP3 inflammasome is associated with numerous autoinflammatory, autoimmune, and metabolic diseases including diabetes, Alzheimer's, and arthritis.^43^ Additionally, gain‐of‐function mutations in NLRP3 are associated with cryopyrin‐associated periodic syndrome (CAPS), manifested by recurrent episodes of joint pain, fever, rashes, and conjunctivitis.^44^, ^45^, ^46^ Based on the severity of these symptoms, CAPS is clinically classified into three disease subclasses.^45^, ^46^ Familial cold autoinflammatory syndrome (FCAS) represents the mildest form of the disease, characterized by cold‐induced inflammation and IL‐1β production. Muckle–Wells syndrome (MWS) exhibits an intermediate phenotype, while the neonatal‐onset multisystem inflammatory disease/chronic infantile neurologic cutaneous articular syndrome (NOMID/CINCA) presents the most severe symptoms.

Besides NLRP3, inflammasomes formed by other NLRs and PRRs have also been defined. The NAIP‐NLRC4 inflammasome is activated in response to bacterial flagellin, PrgJ rod protein, and needle subunits of the type 3 secretion system.^47^, ^48^, ^49^ These ligands, which bind specific members of the NAIP family, permit NAIP‐NLRC4 interaction. Studies employing purified components have revealed that a single PrgJ‐bound NAIP2 molecule is sufficient to activate NLRC4, which undergoes substantial structural re‐organization, interacting with and activating other NLRC4 molecules to eventually form a 10‐ to 12‐spoke wheel or disk‐like structure.^48^, ^49^ On the other hand, the inflammasome assembled by AIM2, a PYHIN (pyrin and HIN domain containing) family member, senses the cytosolic presence of dsDNA irrespective of its origin.^50^, ^51^, ^52^, ^53^ The electrostatic interaction between dsDNA and HIN domain relieves the autoinhibitory state of AIM2, facilitating inflammasome activation. The inflammasome is also assembled by pyrin, encoded by the *MEFV* gene.^54^, ^55^ The pyrin inflammasome is activated upon sensing RhoA inactivation by bacterial toxins, including *Clostridium difficile* toxin B and the *Vibrio cholerae* toxin.^56^ Additionally, gain‐of‐function mutations in the *MEFV* gene cause an autoinflammatory disease, familial mediterranean fever (FMV), and macrophages harboring the mutations have revealed increased Caspase‐1 activation and IL‐1β secretion.^57^

In recent years, lipid metabolism has been shown to play pivotal roles in the assembly and activation of the NLRP3 inflammasome.^58^ Lipids play important physiological roles, the most important of which are maintaining the structural integrity of cellular membranes and regulating energy metabolism.^59^ Additionally, lipids have the potential to control immune activity in various ways, including by defining the functional characteristics of various organelles through membrane and protein trafficking between them, the formation of lipid rafts, and by contributing to the activation of transcription factors, as well as serving as second messengers.^60^, ^61^, ^62^ Lipids can be derived from both exogenous and endogenous sources. Exogenously, lipids are obtained from dietary sources via uptake through the LDL receptor, the scavenger receptor, and by binding to CD36 and fatty acid binding proteins. However, when nutrient conditions are limiting, lipids can also be synthesized de novo through the process of fatty acid synthesis or the mevalonate pathway.^63^ The biosynthesis of lipids (lipogenesis) is regulated by the sterol regulatory element‐binding protein (SREBP) family of transcription factors in a sophisticated manner.^64^ Intriguingly, an excess of lipids is cytotoxic. Therefore, any free fatty acids must be promptly utilized or directed toward triglyceride synthesis and stored as lipid droplets. The accumulation of cellular lipids results in the activation of transcription factors, PPARs, and LXRs.^65^ The two transcription factors regulate the expression of transporters such as ABCA1 and ABCG1 for effective lipid efflux.^66^ On the contrary, stored lipids are broken down by lipolysis.^67^ Lipolysis, or lipid catabolism, involves the breakdown of fatty acids to generate cellular energy through β‐oxidation in the mitochondria and the TCA cycle.

Dysregulated lipid metabolism can profoundly alter immune functions, serving as an instigator of several chronic diseases. This includes inflammation driven by the inflammasome effector cytokine IL‐1β within plaque‐laden arterial walls during atherosclerosis and the impairment of insulin signaling during diabetes. Consequently, lipid metabolism and inflammation constitute pivotal components in the progression of chronic diseases. In this review, I highlight the recently discovered fundamental roles of lipids in NLRP3 activation to provide a framework for understanding the mechanisms that link lipid metabolism to inflammasome activation. Comprehending these mechanisms will offer insights into how lipids calibrate inflammasome activation to maintain homeostasis in health and disease.

## 2 LIPID BIOSYNTHESIS AND INFLAMMASOME ACTIVATION

### 2.1 SREBP and SCAP regulate lipid biosynthesis

Lipid biosynthesis is tightly regulated due to the multifaceted roles lipids play in cellular functions. The synthesis of lipids relies on two major transcription factors, SREBP1 and SREBP2. While SREBP1 is predominantly activated by insulin and nutritional signals, transcribing genes involved in fatty acid biosynthesis, SREBP2 regulates the expression of genes involved in cholesterol biosynthesis and is activated in response to changes in the cellular levels of cholesterol.^68^ SREBPs are localized and retained in the ER membranes by interacting with SREBP cleavage‐activating protein (SCAP), which, when bound to cholesterol attains a conformation suitable for forming a complex with another ER protein, Insig1 (insulin‐induced gene 1).^64^ This prevents the translocation of SCAP‐SREBP2 to the Golgi for proteolytic activation under cholesterol‐rich conditions. By contrast, when cholesterol is limiting, sterols dissociate from SCAP, thereby diminishing the affinity between SCAP and Insig1. Once separated, Insig is no longer stable and undergoes ubiquitination and rapid degradation.^69^, ^70^ The SCAP‐SREBP complex then detaches from the ER and translocates to the Golgi, packaged in COPII (coat protein complex II) vesicles, where SREBPs are sequentially cleaved to their mature and functional forms by site‐1 protease (S1P) and site‐2 protease (S2P).^68^, ^71^ The processed N‐terminal domain of SREBP translocates to the nucleus elevating the expression of genes involved in lipid biosynthesis, as well as Insig. When bound to Insig under cholesterol‐rich conditions, the SCAP‐SREBP2 complex cannot be incorporated into COPII vesicles preventing the transcription of SREBP target genes.^72^ This feedback mechanism allows cholesterol to regulate its synthesis. The binding of SCAP‐SREBP2 to Insig can also be regulated by oxygenated derivatives of cholesterol, including 25‐hydroxycholesterol (25‐HC).

### 2.2 Cholesterol biosynthesis, trafficking, and accumulation promote inflammasome activation

**FIGURE 1:**
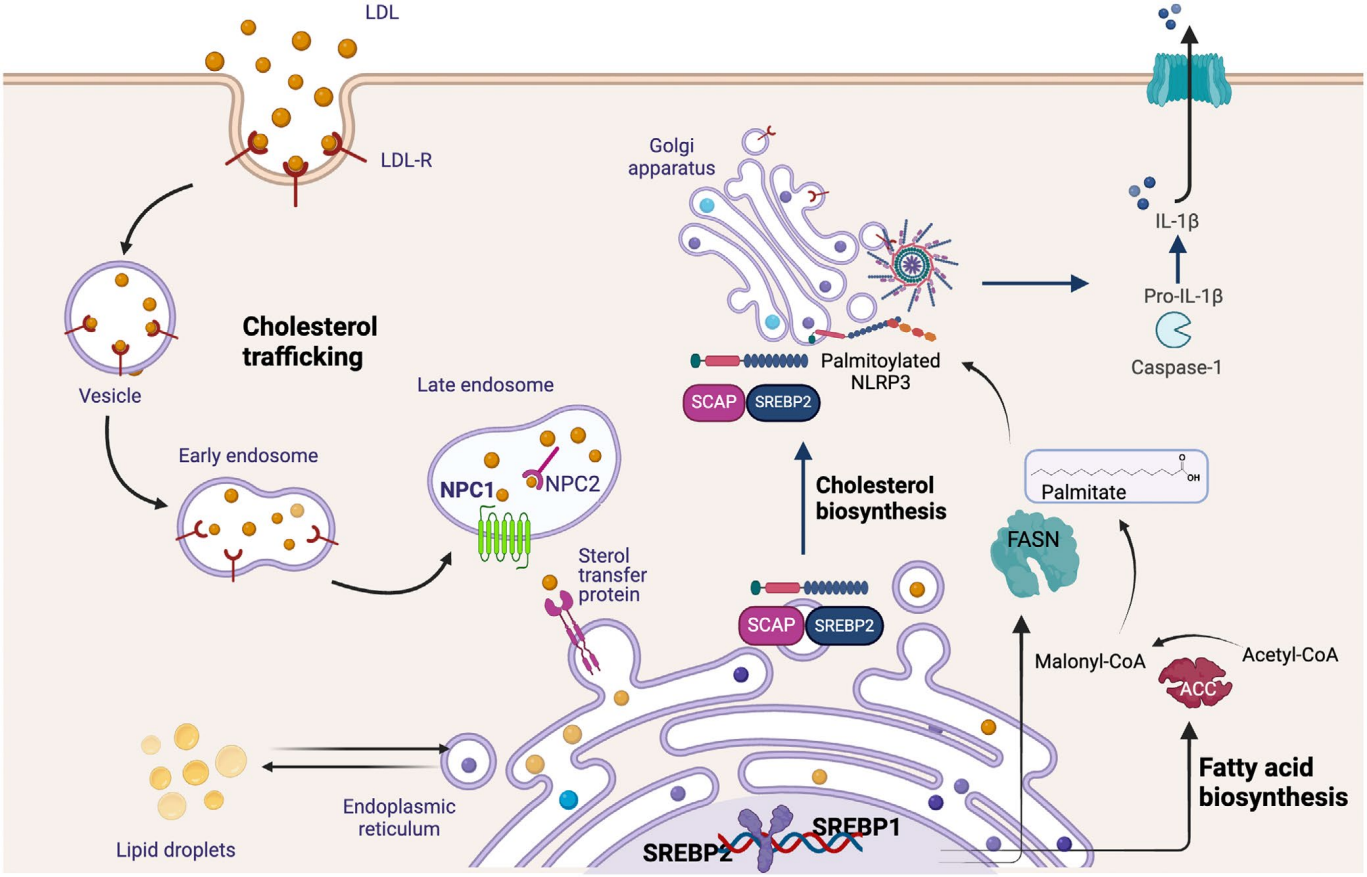
NLRP3 inflammasome activation is regulated by lipid biosynthesis and cholesterol trafficking. Cholesterol obtained through exogenous sources such as low‐density lipoproteins (LDL) is endocytosed via the LDL receptor. Once within the late‐endosomal compartment, hydrolysis of cholesterol esters makes available free cholesterol which exits the compartment through the use of lysosomal membrane resident cholesterol transporter NPC1. This cholesterol efflux to the ER is important as inhibition of either NPC1 or ER cholesterol levels results in dampened inflammasome activation. NLRP3 has also been shown to form a ternary complex with SCAP‐SREBP2 at the ER. Under low‐cholesterol conditions, the complex translocates to the Golgi compartment close to mitochondrial cluster for inflammasome assembly. The translocation to the Golgi compartment is additionally dependent on fatty acid biosynthesis and the production of downstream metabolite palmitate. Palmitate conjugates to NLRP3 in a process known as palmitoylation increasing the affinity of NLRP3 for membranes. Inhibition of a critical enzyme in this pathway, fatty acid synthase (FASN), or palmitoylation blunts the translocation of NLRP3 to the trans Golgi compartment and thus inflammasome activation. ACC, acetyl CoA carboxylase; FASN, fatty acid synthase; LDL‐R, low‐density lipoprotein receptor; NPC1, Niemann–Pick type C1; SCAP, SREBP cleavage‐activating protein; SREBP, sterol regulatory element‐binding protein.

Cholesterol biosynthesis and trafficking have been demonstrated to be pivotal for NLRP3 inflammasome activation. The assembly and translocation of the SCAP‐SREBP2 complex from the ER to the Golgi apparatus involves NLRP3, forming a ternary complex.^73^ Intriguingly, NLRP3 interacts with both SCAP and SREBP2 through its NACHT domain. While the specific residues of interest were not examined, NLRP3 was observed to interact with the C‐terminal regulatory domain of SREBP2. The formation of the NLRP3 and SCAP‐SREBP2 complex led to spontaneous inflammasome activation when cholesterol biosynthesis was enforced (Figure 1). Conversely, exposure to SCAP‐SREBP2 inhibitors or endogenous sterols suppressed inflammasome activation.^73^ Strikingly, both the inflammasome priming and activation signals promote NLRP3 proximity to SCAP‐SREBP2 as observed by microscopy. However, re‐localization to the Golgi (adjacent to mitochondrial cluster) was predominantly triggered upon exposure to the activation signal.^73^ These studies were further validated by biochemical approaches revealing NLRP3 expression in both the Golgi and mitochondrial fractions upon nigericin stimulation. Intriguingly, NLRP3 was found close to COPII protein Sec23A implying that the complex exploited COPII vesicles for NLRP3 translocation.^73^ Once at the Golgi, the cleavage of SREBP2 by S1P was sufficient to release NLRP3 from the complex, thereby facilitating inflammasome assembly. Consequently, experiments with S1P‐deficient BMDMs resulted in reduced IL‐1β secretion. Conversely, exposure to brefeldin A, which causes Golgi to collapse into ER resulting in constitutive SREBP2 processing by S1P, induced spontaneous IL‐1β maturation.^73^, ^74^ These findings underscore the critical role of cholesterol biosynthesis and vesicular trafficking in NLRP3 inflammasome activation.

The regulation of NLRP3 does not rely on SREBP2 transcriptional activity but is exclusively dependent on SCAP‐SREBP2 escort activity. However, important questions remain unanswered. The implications of excessive NLRP3 translocation to the Golgi during lipogenesis and, conversely, unwanted SREBP2 processing during inflammasome activation remain unclear. It has been proposed that lipogenesis, during NLRP3 activation and resulting pyroptosis, may serve to preserve the plasma membrane integrity should IL‐1β alone be sufficient to remove the threat. Though, unwarranted lipogenesis is cytotoxic and may activate the AIM2 inflammasome by compromising mitochondrial integrity.^75^ More importantly, the need for a rapid inflammatory response to imminent threats necessitates inflammasome activation mechanisms that operate independently of lipogenesis and other physiological processes. This is crucial because many pathogens can trigger inflammasome activation without altering cholesterol levels or biosynthesis, although a downstream activation of SREBP by Caspase‐1 has been proposed during bacterial infection.^76^

The nature and localization of cholesterol within cellular organelles trigger distinct mechanisms for NLRP3 inflammasome activation and may also activate different inflammasomes.^77^ In its crystalline form, cholesterol is established as the primary inflammatory stimulus and its presence directly correlates to the recruitment of inflammatory cells and inflammasome activation during atherogenesis.^78^ Indeed, several endogenous and environmental stimuli, in their crystal form, have also been implicated in NLRP3 activation in pathological conditions.^34^, ^35^, ^79^ The crystal‐independent form of cholesterol is also known to activate the NLRP3 inflammasome. The lipoproteins taken up via the LDL‐receptor endocytic pathway are routed to endosomes/lysosomes where cholesterol esters are hydrolyzed by lipases (Figure 1). Cholesterol is heterogeneously distributed within cellular membranes with the majority localized in the plasma membrane. To meet the demands of organelle membranes, cholesterol must exit the lysosomes via cholesterol transporter NPC1.^64^ In *Npc1*‐deficient cells, impaired lysosomal cholesterol efflux disrupts the AKT–mTOR pathway as a direct consequence of altered plasma membrane composition. Additionally, defective cholesterol transport from the NPC1‐positive compartment to the ER reduces inflammasome activation.^80^ These findings are mirrored in cells where ER cholesterol levels were acutely depleted by statins, resulting in reduced IL‐1β secretion. Statins have also been shown to inhibit SREBPs and inflammasome activation during SARS‐CoV‐2 infection.^81^ Yet, statins may also trigger SCAP‐SREBP2 translocation to the Golgi in complex with NLRP3, thereby promoting inflammasome activation.^73^ Notably, the latter findings are somewhat surprising given that statins, which are widely used to lower cholesterol levels, are well‐established anti‐inflammatory agents. On the contrary, statins are also associated with increased risk of diabetes and myopathy so their precise roles may vary with cell type and disease. Strikingly, a related protein NPC1‐like 1 (NPC1L1), which lacks the endosomal targeting motif, has also been shown to promote inflammasome activation.^82^ In mice and zebrafish fed a high‐cholesterol diet, NPC1L1 at the plasma membrane facilitated cholesterol uptake and promoted myeloid cell accumulation through inflammasome‐dependent IL‐1β production.^83^ This highlights the role of NPC1 and NPC1L1 in inflammasome activation, despite their localization in different subcellular compartments. Remarkably, mutations in the human *NPC1* gene are linked to a lipid storage disorder, Niemann–Pick disease. It would be valuable to investigate and systematically distinguish the impact of defective inflammasome activation in these patients from the other severe symptoms of NPC disease.

Overall, these findings implicate sterol synthesis and trafficking as critical factors linking lipid homeostasis to inflammasome activation. Moreover, these findings also outline how sterol defines the cellular localization of NLRP3. Priming of the inflammasome has been associated with NLRP3 localization to the ER. While this initial localization may occur independently, the ER cholesterol pool is critical for subsequent inflammasome activation. However, the precise role of ER cholesterol remains uncertain; it may either support the accurate conformation of NLRP3 or assist in its packaging and transport to the Golgi for inflammasome activation. Regardless, it is tempting to speculate that the NLRP3 inflammasome has the potential to assemble in distinct organelles depending on the nature of upstream stimuli and the lipid composition of cell growth media.

Cholesterol biosynthesis and accumulation have been reported to activate distinct inflammasomes. By employing macrophages lacking cholesterol 25‐hydroxylase (Ch25h), the enzyme that generates 25‐HC by cholesterol hydroxylation, it has been demonstrated that 25‐HC inhibits NLRP3 priming and activation by impeding sterol biosynthesis.^84^ Notably, 25‐HC suppresses cholesterol biosynthesis by directly binding to Insig protein, which retains SREBP2 in the ER. Consequently, *Insig* overexpression in *Ch25h*‐deficient macrophages, and *Scap*‐deficiency in macrophages, both revealed a decrease in NLRP3 activation.^84^ Collectively, the studies discussed above suggest that cholesterol biosynthesis and trafficking are key factors in NLRP3 inflammasome activation. Additionally, the role of 25‐HC has been extended to include the regulation of AIM2 inflammasome activation.^75^ The inability of *Ch25h*‐deficient macrophages to regulate physiological sterol biosynthesis results in cellular cholesterol accumulation leading to disruption of mitochondrial membrane integrity, release of mtDNA into the cytoplasm, and activation of the AIM2 inflammasome.^75^ Accordingly, cholesterol loading by employing methyl β‐cyclodextrin, similarly, activated the AIM2 inflammasome. In contrast, it has been proposed that 25‐HC plays a relatively modest role and the majority of the feedback inhibition of cholesterol biosynthesis occurs through direct binding of cholesterol to SCAP.^85^ Moreover, *Ch25h*‐deficient mice show normal cholesterol metabolism.^86^ Furthermore, other studies have reported that cholesterol depletion by methyl β‐cyclodextrin may either promote or has no role in NLRP3 activation,^73^, ^80^ demonstrating how balance in cholesterol depletion, biosynthesis, and accumulation may influence inflammasome activation in contrasting ways.

### 2.3 Fatty acid biosynthesis and palmitoylation promote NLRP3 inflammasome activation

While a plethora of research has examined the roles of cholesterol biosynthesis, trafficking, and accumulation in inflammasome activation, the roles of fatty acid biosynthesis remained relatively unexplored. Fatty acid biosynthesis proceeds through a complex but an elegant mechanism in which the whole process of lipogenesis largely takes place on a single holoenzyme, fatty acid synthase (FASN), consisting of six catalytic sites.^87^ The reaction begins by utilizing as a substrate acetyl‐CoA, primarily derived from carbohydrate metabolism, and converting it into malonyl‐CoA.^88^, ^89^, ^90^ Malonyl‐CoA serves as a two‐carbon donor in each cycle of a series of reactions catalyzed by FASN, elongating the growing fatty acid chain. The resulting product palmitate, a 16‐carbon saturated fatty acid, serves as a precursor to long‐chain fatty acids (Figure 1). While fatty acids contribute to the structural requirements of the cell and maintain energy homeostasis, palmitate can also directly and reversibly conjugate to the cysteine residues of target proteins in a process known as palmitoylation.^91^, ^92^ Protein palmitoylation is fundamental to cellular functions as it increases proteins hydrophobicity allowing them to tether to membranes, thus defining their localization and function.

Earlier studies demonstrated the involvement of FASN in inflammasome priming by regulating the production of acetoacetyl‐CoA, which contributed to the plasma membrane lipid raft formation.^89^ Palmitate has also been shown to directly act as a second signal and activate the NLRP3 inflammasome in LPS‐primed macrophages and DCs resulting in increased 1β and IL‐18 production.^93^ However, this required exposure to high concentrations of BSA‐conjugated palmitate (up to 500 μM) for up to 24 h to observe the effect. Similarly, the effect of palmitate on inflammasome priming and activation has also been observed in sebocytes, the major cell type in sebaceous glands.^94^ Thus, while a causal relationship between lipid metabolism and inflammasome activation has been demonstrated, direct evidence of the role of fatty acid biosynthesis in inflammasome activation remains unexplored.

Very recently, it has been demonstrated that fatty acid biosynthesis and the direct product palmitate are critical to NLRP3 localization and inflammasome activation.^95^ It was observed that inhibiting either FASN or palmitoylation after the priming step attenuated inflammasome activation. Intriguingly, a role for multiple palmitoylation sites has been reported in NLRP3 activation, demonstrating tight regulation of the NLRP3 inflammasome by palmitoylation.^95^, ^96^, ^97^, ^98^ Consequently, FASN inhibition or mutagenesis of the NLRP3 palmitoylated residue resulted in diminished NLRP3 palmitoylation (Figure 1). Mechanistically, TLR priming resulted in NLRP3 Cys898 palmitoylation, while cells overexpressing mutant NLRP3 Cys898 exhibited reduced inflammasome activation compared to NLRP3 WT cells. Together with published literature, these studies revealed that FASN contributes to both inflammatory signaling and inflammasome activation. Interestingly, GSDMD has also been recently shown to be palmitoylated at the Cys191/192 (human/mouse) site regulating the membrane translocation of the N‐terminal fragment of GSDMD, although palmitoylation itself was not found to be important for GSDMD processing.^99^, ^100^ Notably, the effects of pharmacological inhibition of palmitoylation could be rescued by exogenous palmitate, resulting in restoration of inflammasome activity. Besides Cys898 site, the roles for palmitoylation at Cys126 and Cys837/838 have also been reported in inflammasome activation.^95^, ^96^, ^97^ This phenomenon, which is akin to the presence of multiple phosphorylation and ubiquitination sites in NLRP3, highlights how different palmitoylation sites likely regulate at different stages of inflammasome assembly and activation. Indeed, while palmitoylation at Cys837/838 is important for NLRP3‐NEK7 interaction, Cys126 and Cys898 sites allow NLRP3 translocation to the *trans* Golgi network (TGN) compartment. It is worth noting that NLRP3 has been separately reported to associate with the dispersed TGN compartment through a polybasic sequence in NLRP3.^101^ Moreover, it has been proposed that NLRP3 association to membranes is essential in promoting the NLRP3 double‐ring cage structure for activation.^102^ While polybasic sequence may result in membrane association, it is characteristically considered inadequate for robust membrane attachment and a second lipid anchor is typically needed to increase membrane affinity. This is best exemplified by Ras and heterotrimeric G proteins which require at least two different anchors for stable membrane attachment.^103^, ^104^ Overall, palmitoylation is an important mechanism that directly provides the anchor for inflammasome to assemble on the TGN compartment. These findings directly implicate lipids, and more crucially the process of fatty acid biosynthesis, in calibrating inflammasome activity.

## 3 NLRP3 ACTIVATION IS MODULATED BY PI4P EFFECTORS AND FATTY ACYL CHAIN COMPOSITION

Phosphatidylinositols, a group of lipid molecules that play crucial roles in cellular signaling, have also been recently implicated in NLRP3 inflammasome activation. Structurally, PIs are composed of a glycerol backbone, two fatty acid chains, and an inositol ring bound to a phosphate group.^105^ As a fundamental characteristic, the inositol ring of PI can be phosphorylated at different positions leading to the formation of various phosphoinositides, which are important in a variety of processes.^105^ The hydrolysis of PIP2, in particular, results in the production of important second messengers IP3 and DAG, which have been associated with inflammasome activation. Moreover, PI4P, which is predominantly localized in the Golgi, has also been implicated in modulating inflammasome assembly.^101^ PI4P accumulation in the Golgi resulted in NLRP3‐GFP translocation to the Golgi, where NLRP3 formed multiple puncta before oligomerizing with ASC. Strikingly, the dispersion of TGN is the earliest event which is required for NLRP3 activation in response to diverse NLRP3 stimuli. This recruitment of NLRP3 to the TGN compartment relied on NLRP3 polybasic region forming ionic bonds with dispersed TGN PI4P pool.^101^ However, palmitoylation may also contribute to robust association to the Golgi.^95^ DAG, which is primarily located in the cytoplasm, has also been shown to translocate to the Golgi upon NLRP3 activation. Remarkably, disruption of the Golgi structure by brefeldin A diminished NLRP3 activation.^106^ NLRP3 activation in this study was shown to require NLRP3 Ser293 phosphorylation by DAG effector protein kinase D. Importantly, protein kinase D inactivation prevented inflammasome activation but NLRP3 oligomerization on mitochondria‐associated ER membranes (MAMs) proceeded unrestricted, suggesting that Ser293 phosphorylation by protein kinase D allowed NLRP3 to leave MAMs to form NLRP3 assembly.^106^ These studies are clinically relevant as pharmacological inhibition of protein kinase D in cells from CAPS patients also reduced inflammasome activation. The composition of the two fatty acids attached to PI may also independently regulate inflammasome activation. An increase in linoleic acid as part of the two PI acyl chains (which are otherwise composed of saturated stearic acid (18:0) at *sn*‐1 position and unsaturated arachidonic acid (20:4) at *sn*‐2 position) resulted in increased degradation of TIRAP. This elevated the NLRP3 Ser291 phosphorylation through the PGE2‐induced protein kinase A pathway, which is inhibitory to inflammasome activation.^107^ These data demonstrate that altered PI acyl chain configuration results in elevated PGE2/PKA signaling, which phosphorylated NLRP3 and blunted inflammasome activation. Notably, the dysregulation of PI metabolism is associated with various diseases including cancer, diabetes, and neurodegenerative disorders. Therefore, examining how PI metabolism in these diseases affects NLRP3 activation may offer insights to improve therapeutics for these conditions.

## 4 LIPID PEROXIDATION AND REGULATION OF PYROPTOSIS

Cellular inflammatory responses are intricately regulated by cytokines, growth factors, and lipids at infection or injury sites. A complex mixture of oxidized lipids released from dying cells, in particular oxidized 1‐palmitoyl‐2‐arachidonoyl‐sn‐glycero‐3‐phosphocholine (oxPAPC), has been shown to bind to CD14 and undergo endocytosis. OxPAPC directly binds to caspase‐11, activating the NLRP3 inflammasome.^108^ Surprisingly, this occurred without the concomitant pyroptosis resulting in a hyperactivation phenotype. Moreover, the findings were evident in both cell cultures and in mice, where increased IL‐1β was observed without the lethal sepsis typically induced by LPS. These findings underscore the pivotal roles of lipids in fine‐tuning immune responses to infectious and sterile stimuli. Other endogenous lipids have also been shown to bind to NLRP3 but contrastingly inhibit the NLRP3 inflammasome. 4‐hydroxynonenal (4‐HNE), a major endogenous product of lipid peroxidation, binds to NLRP3 inhibiting the NLRP3‐NEK7 interaction.^109^ This activity of HNE proceeded independently of Nrf2 and NF‐kB signaling, two established pathways by which HNE regulates inflammation. Consequently, administration of exogenous HNE or increasing endogenous HNE levels by impeding glutathione peroxidase 4 (GPX4), which attenuates HNE formation, inhibited inflammasome activation during acute lung injury and sepsis in mice. Conversely, higher HNE concentrations are associated with increased IL‐1β and IL‐18 production in human retinal pigment epithelial cells.^110^, ^111^ In agreement, myeloid cell deficiency in *Gpx4* increased caspase‐11‐dependent pyroptosis and septic lethality, which were reversed upon administration of antioxidant vitamin E.^112^ Remarkably, *Gpx4* deficiency is also associated with elevated apoptosis, necroptosis, and ferroptosis.^113^, ^114^, ^115^, ^116^ Consequently, GPX4 is protective in several inflammatory and metabolic diseases.^117^, ^118^ These studies highlight the differences between acute and chronic GPX4 depletion and reveal crucial insights into the regulation of inflammasome by lipid peroxidation.

## 5 LIPID UPTAKE AND ACCUMULATION ARE ASSOCIATED WITH NLRP3 ACTIVATION

Lipids are complex molecules consisting of a head group that is esterified to hydrophobic tails consisting of either fatty acyl chains or sphingoid base. The uptake of lipids is critical for modulating innate immunity and inflammation by influencing various inflammatory functions. Lipid uptake and trafficking are mainly mediated by CD36, the LDL receptor and fatty acid binding proteins.^119^ CD36 is a cell surface receptor that delivers mainly long‐chain fatty acids and oxidized LDL to cells. This involves several receptors that play critical roles in maintaining lipid homeostasis and are often implicated in inflammasome activation.

CD36, a scavenger receptor involved in fatty acid uptake, recognizes oxidized LDL and nucleates it into cholesterol crystals facilitating inflammasome activation.^120^ The fatty acid crystal formation may be enabled by acyl‐CoA synthetase 1 (ACSL1). Consequently, ACSL1‐deficient macrophages exhibited reduced fatty acid crystal formation, lysosomal damage, and IL‐1β secretion.^121^ Remarkably, scavenger receptor class B type 1 (SR‐B1), which is the primary receptor for HDL, has recently been shown to exhibit affinity for silica particles mediating silica internalization and inflammasome activation.^122^ Independently, lipid accumulation is also associated with increased inflammasome activation. In obese mice and in hepatocytes exposed to fatty acids, expression of CCN1, a secreted matricellular protein with varied cellular functions, increased free fatty acid accumulation resulting in heightened NLRP3 activation and pyroptosis by interacting with the integrin α_5_β_1_ receptor.^123^ In a mouse model of steatohepatitis‐related cardiomyopathy, accumulation of free cholesterol was associated with increased RNA and protein expression of NLRP3, caspase‐1 and IL‐1β in liver and heart suggesting a role for inflammasome priming.^124^ Similarly, uptake of cholesterol from the intestine into enterocytes by NPC1L1 increases inflammation in an IL‐1β‐dependent manner. Consequently, inhibition of cholesterol binding/uptake via NPC1L1 by treatment with ezetimibe abolished inflammatory functions.^83^ Together, these findings highlight the varied mechanisms by which lipid uptake and accumulation expand inflammasome activation.

## 6 LIPIDS AND INFLAMMASOMES IN METABOLIC DISORDERS

Fatty acids, by modulating inflammasome activation through signaling mechanisms, have also been linked to disease pathogenesis. The exposure of LPS‐primed macrophages and dendritic cells to saturated fatty acids such as palmitate and stearic acid leads to enhanced secretion of IL‐1β. This results in the inhibition of the AMP‐kinase/ULK‐1 pathway and increased insulin resistance as indicated by the phosphorylation of insulin receptor substrate‐1 (IRS1) on serine/threonine residues, which impairs insulin signaling.^93^ The activation caused by saturated fatty acids can be inhibited in the presence of the unsaturated fatty acid, oleic acid.^125^ This inhibitory effect on inflammasome activation is also mimicked by arachidonic acid which inhibited the NLRP3 inflammasome by reducing JNK activation.^126^

Inflammation has been established as a primary instigator of diabetes, and TNF‐α neutralization has been shown to improve insulin sensitivity.^127^ However, contributions of the NLRP3 inflammasome to disease pathogenesis have also been appreciated in recent years. Exposure to saturated fatty acids can promote inflammasome activation through the synthesis of ceramides and DAGs.^128^ Ceramides, particularly, have been associated with the inflammatory response in diabetes where they trigger Caspase‐1 activation and IL‐1β secretion impairing insulin sensitivity.^129^, ^130^ Remarkably, obesity is associated with adipose tissue hypoxia and cell death.^131^, ^132^ Consequently, several DAMPs released during cell death may also activate the inflammasome signifying the complexity of precisely identifying endogenous metabolites in instigating diabetes.

On the other hand, contradictory roles for IL‐18 have been reported. Increased IL‐18 levels in obese individuals have been associated with insulin resistance.^133^ These findings are mirrored in high‐fat diet‐induced obese mice which exhibit increased serum IL‐18 and caspase‐1 activation in adipose tissue and liver.^134^ In contrast, IL‐18 administration is associated with reduced weight gain and IL‐18‐deficiency increases insulin resistance.^135^, ^136^ It is possible that IL‐18 may initially balance lipid homeostasis on a high‐fat diet, but chronically high levels could lead to IL‐18R downregulation leading to higher IL‐18 levels in the serum of obese individuals.^137^, ^138^ Alternatively, IL‐18 production during metabolic syndrome may be regulated by different upstream pathways. Indeed, a protective role for NLRP1‐dependent IL‐18 has been demonstrated during adiposity and metabolic syndrome.^139^ Consequently, mice with an NLRP1 activating mutation exhibit resistance to diet‐induced metabolic dysfunction.^139^ These studies highlight the complex and sometimes contradictory roles of lipids and inflammasome‐dependent cytokines in metabolic disorders.

## 7 CONCLUSIONS AND FUTURE PERSPECTIVES

The ability of the NLRP3 inflammasome to interact with a variety of metabolic signals emphasizes its function as a sensor of cellular homeostasis. Recent investigations have elucidated that lipids assume critical roles in the regulation of the NLRP3 inflammasome. Research has emphasized the complex mechanisms through which lipid biosynthesis, transport, and lipid‐derived metabolites regulate inflammasome activity. The participation of cholesterol and lipid biosynthetic pathways, PI4P and their fatty acyl composition, palmitate, oxysterols, and lipid peroxidation collectively illustrate how lipid metabolism influences inflammasome assembly and activation in varied ways. The emerging understanding reveals that lipid metabolism functions as a pivotal hub for the regulation of the inflammasome, with profound implications for diseases characterized by chronic inflammation and metabolic disturbances, though the manner in which these various processes integrate to modulate NLRP3 activity remains ambiguous. Gaining insight into the intricate equilibrium among lipogenesis, lipid utilization, and lipolysis could facilitate the development of novel therapeutic interventions. Nonetheless, caution is necessary, as disruptions in these pathways may be detrimental and increase related pathologies.

Lipids and the activation of inflammasomes each display well‐established roles in the etiology of numerous diseases. However, their specific contributions to disease mechanisms remain ambiguous. An immediate imperative is to elucidate how alterations in lipid metabolism influence chronic inflammation and the progression of diseases. It is crucial to acknowledge that inflammasome‐dependent cytokines, IL‐1β and IL‐18, exhibit varied roles in a variety of pathological conditions. This presents an additional obstacle in leveraging the advantageous effects of inflammasomes and their downstream cytokines while simultaneously restraining their adverse impacts. Future investigations must elucidate these complex interactions to devise targeted strategies for alleviating inflammatory diseases associated with lipid metabolism. By persistently examining the link between lipid metabolism and inflammasome activation, we can make improvements toward novel therapeutics that tackle the underlying causes of inflammation across a broad spectrum of diseases.

## CONFLICT OF INTEREST STATEMENT

The author declares no competing interests.

## Data Availability

Data sharing is not applicable to this article as no new data were created or analyzed in this study.
